# A Seasonal Fresh Tea Yield Estimation Method with Machine Learning Algorithms at Field Scale Integrating UAV RGB and Sentinel-2 Imagery

**DOI:** 10.3390/plants14030373

**Published:** 2025-01-26

**Authors:** Huimei Liu, Yun Liu, Weiheng Xu, Mei Wu, Leiguang Wang, Ning Lu, Guanglong Ou

**Affiliations:** 1 College of Big Data and Intelligent Engineering, Southwest Forestry University, Kunming 650233, China; liuhuimei@swfu.edu.cn (H.L.); swfuluning@gmail.com (N.L.); 2 College of Forestry, Southwest Forestry University, Kunming 650233, China; atly9708@swfu.edu.cn (Y.L.); azkomorebi0309@163.com (M.W.); olg2007621@126.com (G.O.); 3 College of Landscape and Horticulture, Southwest Forestry University, Kunming 650233, China; wlgbain@126.com

**Keywords:** fresh tea yield estimation, machine learning, uncrewed aerial vehicle, feature selection, multiple regression models

## Abstract

Traditional methods for estimating tea yield mainly rely on manual sampling surveys and empirical estimation, which are labor-intensive and time-consuming. Accurately estimating fresh tea production in different seasons has become a challenging task. It is possible to estimate the seasonal yield of tea at the field scale by using the spatial resolution of 10 m, 5-day revisit period and rich spectral information of Sentinel-2 imagery. This study integrated Sentinel-2 images and uncrewed aerial vehicle (UAV) RGB imagery to develop six regression models at the field scale, which were employed for the estimation of seasonal and annual fresh tea yields of the Yunlong Tea Cooperatives in Yixiang Town, Pu’er City, China. Firstly, we gathered fresh tea production data from 133 farmers in the cooperative over the past five years and obtained UAV RGB and Sentinel-2 imagery. Secondly, 23 spectral features were extracted from Sentinel-2 images. Based on the UAV images, the parcel of each farmer was positioned and three topographic features of slope, aspect, and elevation were extracted. Subsequently, these 26 features were screened using the random forest algorithm and Pearson correlation analysis. Thirdly, we applied six different regression algorithms to establish fresh tea yield models for each season and evaluated their estimation accuracy. The results showed that random forest regression models were the optimal choice for estimating spring and summer yields, with the spring model achieving an R^2^ value of 0.45, an RMSE of 40.38 kg/acre, and an rRMSE of 40.79%. Similarly, the summer model achieved an R^2^ value of 0.5, an RMSE of 78.46 kg/acre, and an rRMSE of 39.81%. For autumn and annual yield estimation, voting regression models demonstrated superior performance, with the autumn model achieving an R^2^ value of 0.42, an RMSE of 70.6 kg/acre, and an rRMSE of 39.77%, and the annual model attained an R^2^ value of 0.47, an RMSE of 168.7 kg/acre, and an rRMSE of 34.62%. This study provides a promising new method for estimating fresh tea yield in different seasons at the field scale.

## 1. Introduction

Tea (*Camellia sinensis* (L.) O. Kuntze) is a perennial crop extensively cultivated in hilly terrains of tropical and subtropical regions, playing a crucial role as an important cash crop in various developing nations, such as China, India, and Kenya [1,2,3]. Tea is rich in antioxidants, including catechins and polyphenols, which exhibit anti-inflammatory, antioxidant, and potential anticancer properties [4,5,6]. Moreover, tea gardens play a pivotal role in the ecosystem, as they function as carbon sinks, effectively sequestering carbon dioxide and mitigating greenhouse gas emissions. In 2022, the Food and Agriculture Organization (FAO) of the United Nations reported that China’s tea production reached 3.181 million tons, accounting for 49.1% of global production (https://www.fao.org/home/en, accessed on 5 December 2023). Therefore, estimating tea production is crucial for optimizing tea garden resource allocation, stabilizing tea prices, and fostering sustainable development within the tea industry.

Traditional tea yield estimation methods primarily rely on manual sampling surveys, field observation, and evaluation based on farmers’ experience [5,7,8]. However, these approaches are labor-intensive and often lack accuracy. In recent studies, remote sensing technology and crop growth models have been used to estimate tea yield. For example, Dania et al. [9] employed the AquaCrop simulation model, developed by FAO, to accurately predict tea crop yield with impressive results (MAE = 0.45 t/ha, MSE = 0.23 t/ha, and RMSE = 0.48 t/ha). However, the utilization of crop growth models necessitates extensive parameter calibration [10], involving precise adjustments for climate, soil, crop characteristics, and agricultural management data. In addition, a large number of studies have shown that vegetation indices, such as the Normalized Difference Vegetation Index (NDVI) and the Enhanced Vegetation Index (EVI), and machine learning models, such as random forest (RF), the Support Vector Machine (SVM), and Lasso, achieve significant effects in accurately predicting the yields of different crops, such as grapes, wheat, potatoes, and tea [11,12,13].

To date, remote sensing data with different spatial resolutions have been widely used to estimate tea yield. Phan et al. [14] used medium-resolution MODIS imagery to monitor and analyze NDVI changes during the tea growing season in Tanuyen, Vietnam, and combined it with climate variables to predict tea yield. Ramzan et al. [15] developed a high-performance model that effectively estimates tea yield by integrating high-resolution Landsat-8 image data and agrometeorological data, employing deep learning techniques along with multiple regression and ensemble learning methods. Notably, the deep neural network model performed best, achieving an R^2^ value of 0.99. Fauziana et al. [16] utilized SPOT-7 satellite data and employed linear spectral mixture analysis (LSMA) technology to establish a tea yield estimation model based on canopy density and NDVI, effectively separating various objects within the tea garden. The results demonstrated the high accuracy of this method in estimating tea yield; however, further improvements in accuracy could be achieved by considering factors such as pruning. Another study aimed to assess the impact of varying spatial resolutions on tea yield estimation in the Pagilaran plantation, India [17]. Tea yield was estimated using Landsat-8 OLI images with a spatial resolution of 30 m and Sentinel-2B images with a higher resolution of 10 m. The findings revealed that the utilization of high-resolution Sentinel-2B imagery resulted in more precise tea yield estimation. Moreover, UAV data are widely applied in precision agriculture due to their flexibility and centimeter-level high resolution. For example, Wang et al. [5] used UAV RGB images to detect the number of tea buds and estimate tea yield through deep learning. He et al. [8] utilized UAV hyperspectral data to analyze the spectral differences in tea canopies before and after picking to estimate the yield of fresh tea in spring.

Numerous regression algorithms for tea yield estimation have been applied in previous studies [18,19,20]. A study [9] used ten different machine learning algorithms trained on multi-source data such as meteorological, soil and crop management data to predict tea yield. Among them, XGBoost algorithms performed best, with an MAE of 0.093 t/ha and an RMSE of 0.12 t/ha. Another study used the dragonfly optimization algorithm (DR) and support vector regression (SVR) to screen climate features and then used the random forest algorithm to predict tea production in Bangladesh, showing excellent performance with a correlation coefficient of 0.993 and an MAPE of 11.95% [21]. In addition, another study utilized MODIS NDVI data and meteorological data from 2009 to 2018, using the random forest algorithm to predict tea production in Tanuyen, Vietnam, achieving a high prediction accuracy with an R^2^ of 0.67 [22]. These studies show that the combination of remote sensing data and machine learning algorithms can significantly improve the accuracy of tea yield prediction and provide strong support for tea garden management.

The role of feature selection in machine learning models has received increasing attention, especially in the processing of high-dimensional data. Through appropriate feature screening, redundant features can be reduced, the prediction ability of models can be improved, and computational complexity can be reduced. In the study of tea yield prediction, feature selection can effectively identify the key factors affecting tea yield and further improve the accuracy of models. In recent years, algorithms such as random forest and XGBoost have shown excellent performance in feature selection [23,24].

The main objectives of this study were to (a) assess the potential for tea yield estimation in various seasons by integrating spectral features derived from Sentinel-2 satellite imagery with topographic features extracted from UAV data at the field scale and (b) verify the field-level accuracy of tea yield estimation models in different seasons.

## 2. Materials and Methods

**Figure 1:**
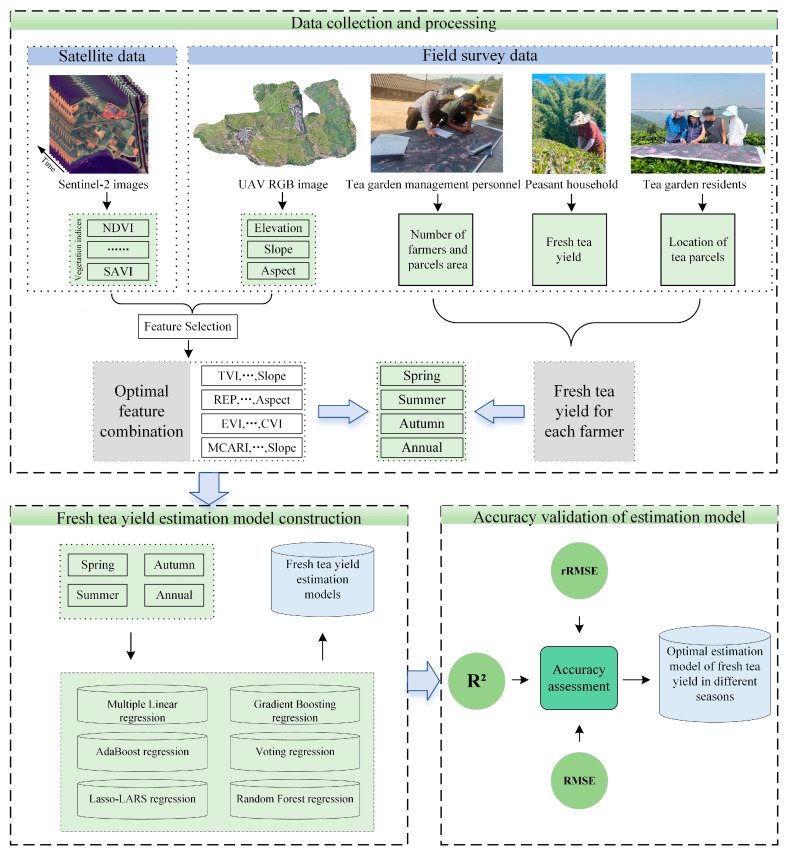
Workflow for tea yield estimation.

The detailed workflow of this study is depicted in Figure 1. Firstly, we utilized Sentinel-2 imagery using the Google earth engine (GEE) platform [25] to extract spectral features for the study area spanning from 2018 to 2022. Simultaneously, the RGB data captured by the UAV were used to generate a digital elevation model, from which slope and aspect features were calculated. In addition, we also obtained information about the location of tea fields, the number of farmers jointly operating the tea farms, the areas of the tea parcels owned by each farmer, and the monthly fresh tea production during the tea growing season from the managers of Yunlong Tea Farm. Secondly, we employed the random forest algorithm to rank the feature importance of spectral features and topographic features in different seasons. In order to eliminate the multicollinearity problem between features, we used Pearson correlation analysis for feature selection. Thirdly, based on the optimal feature combination and yield data, tea yield estimation models suitable for different seasons (spring, summer, and autumn) and whole years were established. Finally, three statistical parameters (the Coefficient of Determination, the Root Mean Square Error, and the relative Root Mean Square Error) were used to evaluate and compare the accuracy of each model in different seasons, and the fresh tea yield estimation model with the highest accuracy was selected.

### 2.1. Study Area

**Figure 2:**
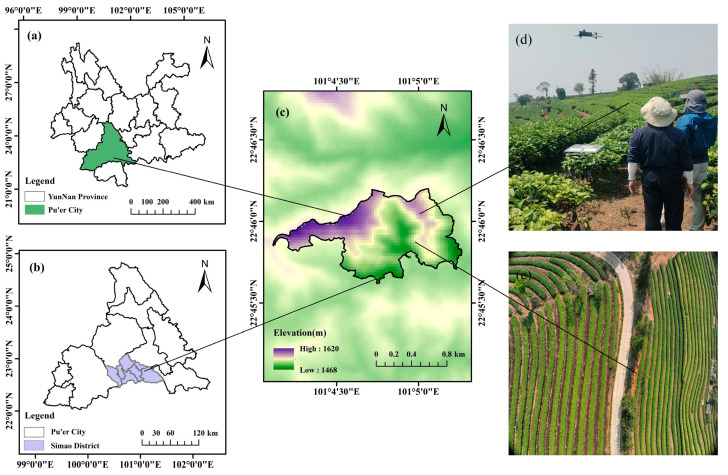
Locations of the experimental sites in this study: (**a**) Yunnan Province; (**b**) Simao District; (**c**) study area (China); (**d**) tea plantation UAV data collection; (**e**) a single UAV image.

The study was conducted in Yixiang Town (22°45′30″ N~22°46′30″ N, 101°4′0″ E~101°5′30″ E), Pu’er city, southwestern Yunnan Province, China (Figure 2). The tea garden area covers approximately 700 acres and is characterized by predominantly mountainous and hilly terrain, with an altitude ranging from 1450 m to 1620 m. The study area has a subtropical plateau monsoon climate, with a mild climate throughout the year, alternating between rainy and dry periods. It has an average annual temperature of around 19 °C, annual rainfall ranging from 770 mm to 1830 mm, and an average frost-free period of 319 days per year. These favorable climate conditions make the region well-suited for tea cultivation [26]. The tea cultivated in this study area belongs to the Camellia sinensis var. assamica variety, with an average tree age of about 27 years. In this study area, due to the differences in tea prices in different seasons, farmers pick tea in different seasons. The tea picked from 1 March to 31 May is called spring tea, the tea picked from 1 June to 31 August is called summer tea, and the tea picked from 1 September to 30 November is called autumn tea.

### 2.2. Data Acquisition and Processing

#### 2.2.1. Field Survey Data

The field survey data for this study were obtained from the Yunlong Cooperative, which consists of 133 tea farmers collectively managing 302 tea field parcels. In this study, 302 tea parcels were picked by skilled tea farmers based on four picking modes: single bud, 1 leaf–1 bud, 2 leaves–1 bud, and 2 leaves. After each picking was completed, the tea farmers sent the freshly picked tea to the cooperative for weighing, and an electronic balance with a sensitivity of 0.01g was used to measure the fresh weight of the buds and young leaves so as to obtain the fresh tea yield data for each farmer from 2018 to 2022. We collated and counted these fresh tea yield data and divided the fresh tea yields into spring yields (March to May), summer yields (June to August), autumn yields (September to November), and annual yields (March to November), according to the growing season of tea in the study area. In addition, through communication with the managers of the Yunlong Tea Farm, we collected relevant information, such as the number of farmers in the tea farm, the tea parcel area of each farmer, and the specific locations of the tea parcels.

Due to factors such as uneven distribution of labor and price fluctuations in the tea market, some farmers did not collect tea in some growing seasons, resulting in null values in yield data. In order to ensure the reliability of the data, this study screened farmers with stable tea-picking activities from 2018 to 2022 and required these farmers to have tea-picking records in spring, summer, and autumn for five years. Finally, we selected the yield data of 40 farmers as the research sample. In order to further improve the accuracy of the data, we used the Mahalanobis distance to eliminate the outliers in the yield data. Therefore, the number of samples was different in different seasons.

#### 2.2.2. Sentinel-2 Images

The growth of crops and the prediction of yield can be effectively achieved through the utilization of diverse spectral bands in satellite data [27]. Sentinel-2 is a second-generation Earth observation satellite operated by the European Space Agency (ESA) [28]. Sentinel-2A and Sentinel-2B, launched in June 2015 and March 2017, respectively, are part of the European Copernicus Plan for observing the Earth’s surface to provide related telemetry services, such as forest monitoring, land-cover change detection, natural disaster management, etc. The multispectral imager (MSI) carried by the Sentinel-2 satellite covers 13 spectral bands with spatial resolutions of 10 m, 20 m, and 60 m. It has a width of 290 km and a 5-day revisit period, making it highly effective for monitoring crop dynamics. For Sentinel-2, we collected Harmonized Sentinel-2 L1C images from 1 March to 31 November 2018 and Harmonized Sentinel-2 L2A images from 1 March to 31 November for the years 2019 to 2022 on the GEE platform. Subsequently, these images were processed for atmospheric correction, cloud masking, median synthesis, and resampled to a 10 m spatial resolution. The Sentinel-2 image data from March to November were divided into three seasons: spring (1 March to 31 May), summer (1 June to 31 August), and autumn (1 September to 31 November).

The construction of vegetation indices can enhance the accuracy of the spectral inversion of vegetation physiological and biochemical parameters and comprehensively reflect the information related to crop growth, biomass, and coverage, which is related to yield. In order to explore the sensitivity of different vegetation indices to the yield estimation model, combined with the growth characteristics of tea trees and previous research results, 7 bands were selected from 13 original reflectance bands, and 23 vegetation indices were calculated. The description and calculation formulae for these indicators are shown in Table 1.

#### 2.2.3. Acquisition and Preprocessing of UAV Images

The four-rotor DJI Phantom 4 RTK (SZ DJI Technology Co., Shenzhen, China) was used from 14 to 15 April 2023 to capture RGB images of the study area. These images had a resolution of 5280 × 3956 pixels and were stored in JPG format. The UAV was autonomously flown by the DJI ground station according to the scheduled route, maintaining an altitude of 60 m and achieving a ground resolution of 2 cm. Before each UAV operation, the ZHD V200 (RTK, GNSS, Guangzhou Hi-Target Navigation Tech Co., Ltd., Guangzhou, China) was used to mark the center point of each tea parcel as a ground control point. These control points were used for geometric correction of UAV images to ensure accurate spatial alignment. To create a comprehensive orthophoto of the entire study area, we set the front-view overlap at 80% and the side-view overlap at 70%. All flights were carried out under clear-sky and low-wind-speed conditions; each flight lasted approximately 30 min.

**Figure 3:**
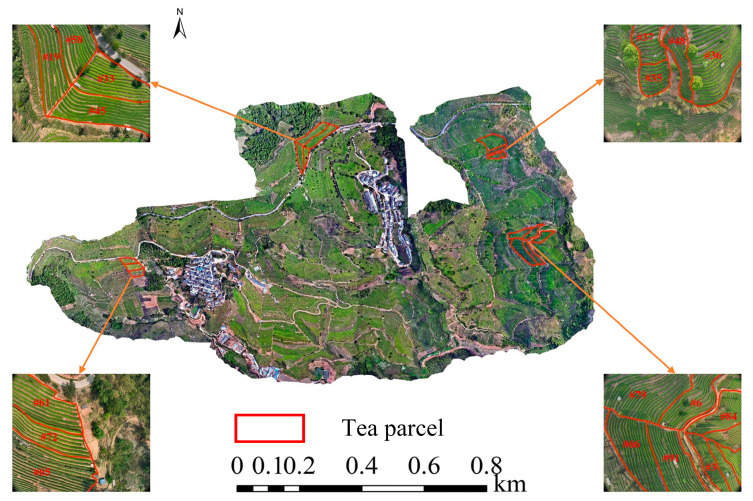
Parcel distribution map of tea field. “#” represents the parcel number.

In order to generate an orthophoto of the study area, we used Pix4Dmapper software (Pix4D SA, Lausanne, Switzerland) to process the UAV images. The main processing steps involved aligning images, matching feature points, and generating a dense point cloud, as well as generating a digital surface model (DSM), a digital terrain model (DTM), and an orthophoto. A DEM is extracted from a DSM through point cloud classification and filtering techniques to exclude nonground features. Finally, ArcMap 10.8 software was used for slope and aspect calculation. The UAV RGB images in this study served as aids for locating and identifying tea garden parcels by extracting digital elevation model (DEM) information (Figure 3).

### 2.3. Methodology

#### 2.3.1. Feature Selection

The extracted features contained redundant information, making feature selection critical. In this section, we explore the application of feature selection methods at different stages of tea growth. Feature selection involves feature importance ranking and Pearson correlation analysis, followed by the addition of suitable features to the regression model.

Feature importance assessment was used to calculate the significance of sample features and quantify their contributions to regression. Random forest (RF) is a machine learning technique that iteratively builds independent decision trees during the training phase [50]. RF can effectively handle a large number of predictor variables and avoid falling into overfitting, as well as being highly resistant to noise in the training data [51]. We used 23 vegetation indices derived from Sentinel-2 satellite data, along with elevation, slope, and aspect generated from UAV RGB data as feature variables. The importance of these variables was then evaluated and ranked using the random forest (RF) algorithm.

The importance score reflected the ratio between the average error and the standard deviation of the variable prediction in each decision tree within the RF algorithm. Furthermore, Pearson correlation analysis was employed to assess the correlation among relevant variables, aiming to mitigate potential multicollinearity issues. In this study, indicators were retained if their feature importance score exceeded 0.05. Additionally, a threshold of 0.7 was assumed for high correlation coefficients using Pearson correlation analysis. If the correlation surpassed 0.7, only indicators with greater significance were retained. Subsequently, indicators with high importance and low correlation were selected for modeling.

#### 2.3.2. Estimation of Fresh Tea Yield Using Regression Models

Machine learning algorithms have demonstrated their exceptional performance across diverse domains, including image and speech recognition, healthcare applications, and autonomous driving [52,53,54]. Machine learning-based methods are increasingly being employed in agricultural sectors for tasks such as crop yield prediction, pest and disease detection, precision irrigation, and soil analysis [9,55,56,57,58]. In this study, we employed one parametric model and five nonparametric models to predict fresh tea yield, including multiple linear regression, AdaBoost regression, Lasso-LARS regression, gradient boosting regression, random forest regression, and voting regression. A concise overview of these regression algorithms is provided below. The specific parameters utilized in each model are presented in Table 2.

Multiple linear regression analysis involves one dependent variable and multiple independent variables, and it is designed to explain the changes in the dependent variable as a function of correlated changes in the independent variables. In crop yield estimation, multiple linear regression is a commonly used method [59,60]. The default parameters for linear regression are utilized in its implementation.

Random forest regression tasks are performed by constructing multiple independent decision trees and integrating their predictions. Each decision tree in a random forest is trained on a randomly selected subsample, effectively mitigating the risk of overfitting. The final regression result in random forests is obtained through averaging or weighted averaging of the predictions from multiple decision trees.

A voting regressor combines predictions from multiple base regressors, each of which is trained on the complete dataset. The different regressors used in this study include gradient boosting, random forest and linear regression models, which are selected for their complementary predictive properties. Subsequently, the voting regressor aggregated their predictions to form a more robust and potentially more accurate final prediction.

Lasso-LARS regression is an optimal choice for high-dimensional datasets, as it effectively combines variable selection and regularization techniques to enhance accuracy and interpretability. By constraining the sum of absolute values of model parameters, this method excludes less significant variables, thereby simplifying the model and improving computational efficiency.

The AdaBoost algorithm, also known as Adaptive Boosting, is an ensemble technique used for regression and classification tasks that combines multiple weak learners, typically decision trees, to create a more robust model. It follows a sequential approach by iteratively adding trees and adjusting instance weights based on their prior performance. Accurate trees are given greater influence, while poorly performing ones have reduced impact, thereby focusing on challenging instances to enhance predictive accuracy.

The gradient boosting regression algorithm iteratively trains a sequence of weak learners to progressively enhance the model’s performance. The fundamental concept behind gradient boosting regression is to construct the subsequent training model based on the residuals of the previous model, thereby gradually diminishing the model’s prediction error based on the given data.

### 2.4. Accuracy Assessment of Estimation Models

All samples from 2018 to 2022 were randomly divided into two parts to construct the regression models. In field-scale experiments, 70% of the dataset was allocated as the training set, while the remaining 30% served as the test set for the spring, summer, and autumn tea yield estimation models and the annual tea yield estimation model. This study compared the performance of six machine learning techniques in integrating spectral and topographic features to estimate tea yield. To assess the performance of the different tea yield estimation models, three distinct statistical parameters were employed: Equation (1), Coefficient of Determination (R^2^), which measures how well the independent variable explains variance in the dependent variable [61]; Equation (2), Root Mean Square Error (RMSE), which quantifies discrepancies between simulated and observed values [62]; and Equation (3), relative Root Mean Square Error (rRMSE) [63]. All data analyses were conducted using the Python programming language, and the formulae for evaluating the model performance metrics are defined as follows:

(1) R2=1−∑i=1n(yi−yi^)2∑i=1n(yi−y¯)2

(2) RMSE=1n−1∑i=1n(yi−yi^)2

(3) $$\mathrm{rRMSE}=\frac{\mathrm{RMSE}}{\bar{y_{i}}}\times 100\%$$

where *n* represents the number of samples during the growing season, $y_{i}$ denotes the measured tea yield, yi^  signifies the predicted tea yield, and $\bar{y}$ represents the average value of the sample.

## 3. Results

### 3.1. Optimal Combination of Features

**Figure 4:**
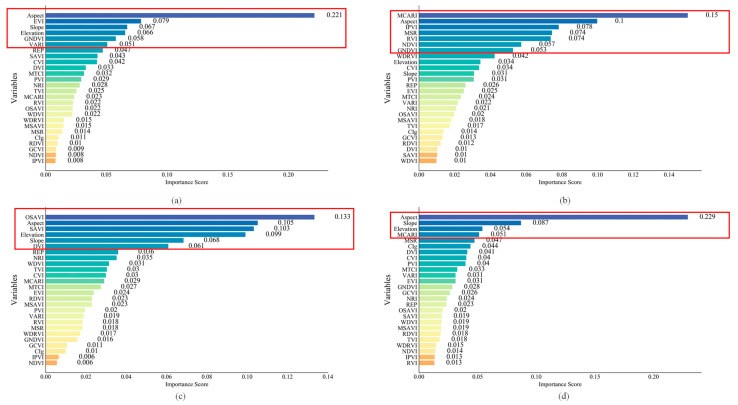
Importance ranking of the features based on field scale: (**a**) spring; (**b**) summer; (**c**) autumn; (**d**) annual. The red box represents the importance score greater than 0.05.

**Figure 5:**
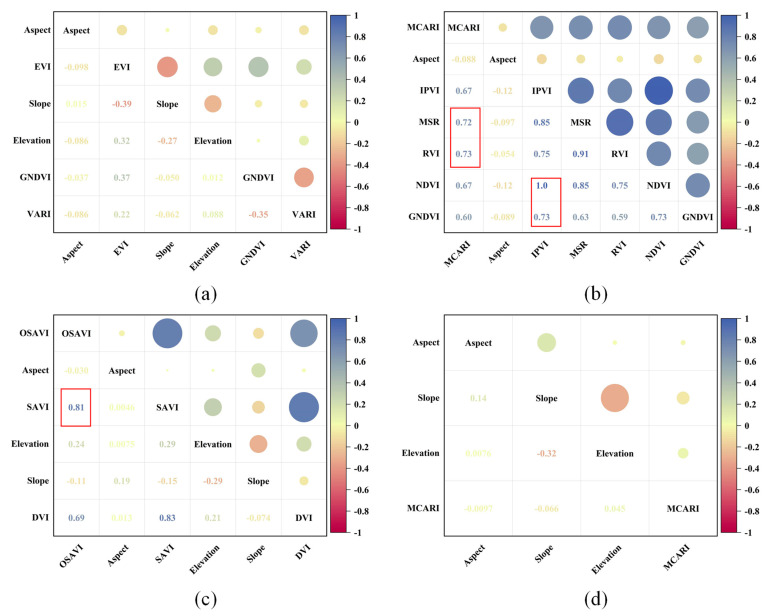
Correlation analysis at the field scale for different seasons: (**a**) spring; (**b**) summer; (**c**) autumn; (**d**) annual.

Based on RF importance ranking and Pearson correlation analysis, the selected features for the spring tea yield estimation model at the field scale included the Enhanced Vegetation Index (EVI), the Green Normalized Difference Vegetation Index (GNDVI), the Visible Atmospherically Resistant Index (VARI), elevation, aspect, and slope (Figure 4a and Figure 5a). For the summer yield estimation model, the chosen indicators were the Modified Chlorophyll Absorption Ratio Index (MCARI), aspect, and the Infrared Percentage Vegetation Index (IPVI) (Figure 4b and Figure 5b). In this experiment, feature variables with high importance and low correlation were preferred for modeling. For instance, in the summer yield estimation model (Figure 4b), MCARI, the Modified Simple Ratio (MSR), and the Ratio Vegetation Index (RVI) were identified as key variables with importance scores exceeding 0.05. However, only MCARI was retained due to its high correlation with MSR (0.72) and RVI (0.73) (Figure 5b). Consequently, MSR and RVI were considered less significant and subsequently excluded. In the estimation of autumn tea yield, aspect, slope, the Optimization Soil-Adjusted Vegetation Index (OSAVI), elevation, and the Difference Vegetation Index (DVI) were selected as variables. Despite its significance as the third most important variable, the Soil-Adjusted Vegetation Index (SAVI) was excluded from the model due to its high correlation with OSAVI (correlation coefficient of 0.81), as shown in Figure 5b. Furthermore, the combination of aspect, slope, elevation, and MCARI demonstrated significant relevance in estimating annual tea production.

### 3.2. Performance of Six Regression Methods in Estimating Fresh Tea Yield

Due to the uneven distribution of the labor force among farmers and the objective factors of tea market price, some farmers’ tea production data for different seasons contained missing values and outliers. To mitigate the impact of these outliers, we performed data cleaning on the collected production data from 2018 to 2022 prior to modeling. After cleaning, the numbers of samples in spring, summer, and autumn and the annual numbers were inconsistent. Then, we randomly divided these samples for different seasons into a training set and a test set according to the ratio of 7:3, which were used to fit six regression models, and the tea yield was estimated by combining the satellite spectral features and topographic features extracted by the UAV. In the experiment based on the field scale, we extracted the spectral characteristics of each farmer’s parcel. The specific method was to calculate the mode of all the pixels in the parcel as the spectral characteristics of the farmer. Similarly, the topographic features were also extracted according to the units of the farmers. We extracted the topographic features of each farmer from the RGB data collected by the UAV and used the mode of all the pixels in the field as the final topographic features for the farmer.

At the field scale, we randomly divided 180 spring tea yield estimation samples into a training set and a validation set according to the ratio of 7:3. Six regression models based on spectral and topographic features were fitted using the training set (n = 126) to estimate spring tea yield, and the accuracy of these models was evaluated using the validation set (n = 54). Finally, we established a multiple linear regression model between the spring tea yield and the selected spring variables, as shown in Equation (4).

(4) $$\mathrm{Yiel}d_{\left(\mathrm{spring}\right)}=\;-0.259\times \mathrm{Aspect}+0.0457\times \mathrm{Elevation}-27.8501\times \mathrm{EVI}+1.7383\times \mathrm{Slope}+19.739\times \mathrm{GNDVI}+23.0356\times \mathrm{VARI}+29.560$$

For the indicators in Equation (4), the relevant definitions and formulae are shown in Table 1.

**Figure 6:**
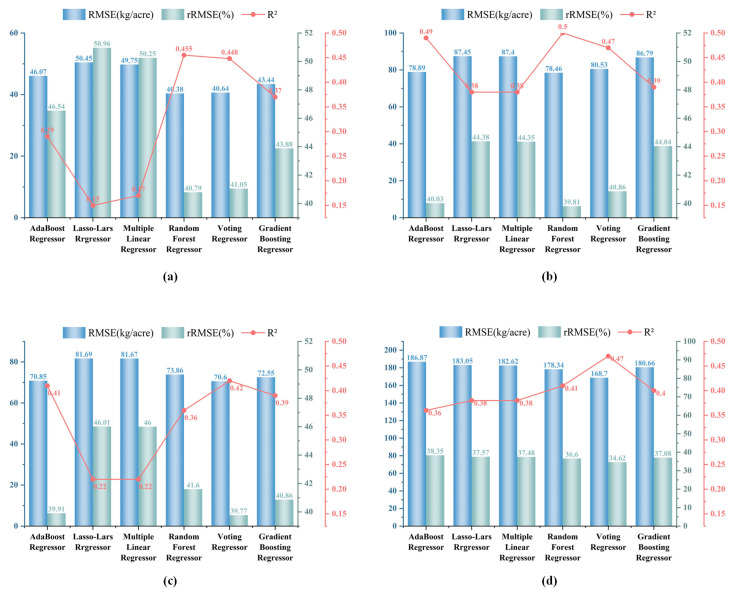
Comparison of fresh tea yield estimation regression model and annual yield estimation regression model for different seasons: (**a**) spring; (**b**) summer; (**c**) autumn; (**d**) annual.

**Figure 7:**
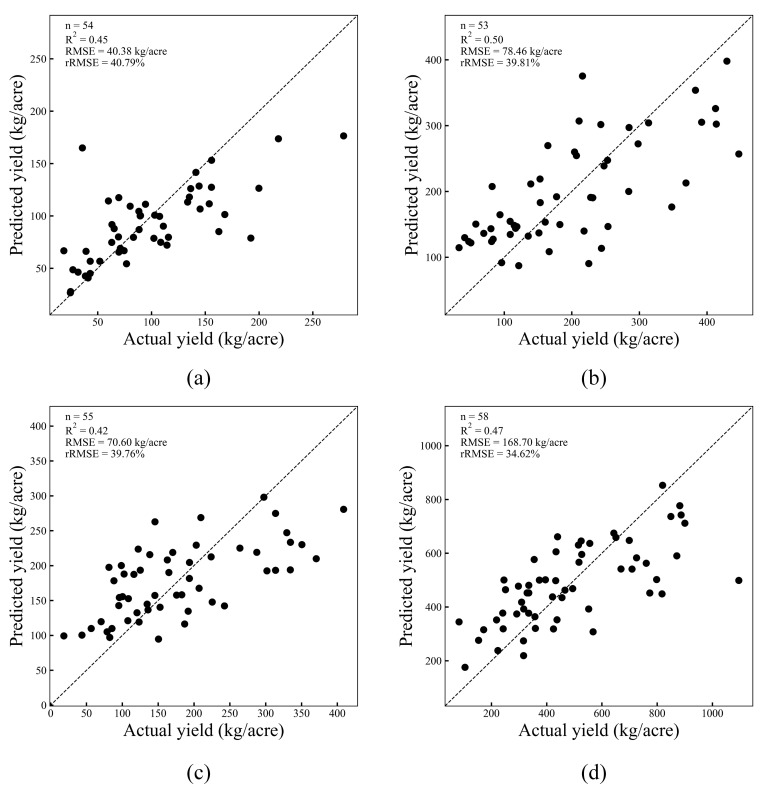
The prediction results for fresh tea yield of the best estimation model for different seasons: (**a**) spring; (**b**) summer; (**c**) autumn; (**d**) annual.

In addition, five other machine learning algorithms were trained using the selected spectral and topographic features as predictors and the yield as the target variable. These algorithms were implemented in Python, and three types of statistical indicators were used for performance evaluation, as shown in Figure 6. By analyzing the results of these regression algorithms, it was concluded that random forest regression performed best in estimating spring tea yield, with an R^2^ value of 0.45, an RMSE of 40.38 kg/acre, and an rRMSE of 40.79% (Figure 7a).

In estimating the yield of fresh tea leaves in summer, we randomly divided 175 samples into a training set (n = 122) and a validation set (n = 53). Based on Equation (5), a multiple linear regression model was constructed to analyze the relationship between summer tea yield and summer characteristics. In the summer yield estimation model shown in Figure 6, R^2^ values ranged between 0.38 (Lasso-LARS) and 0.5 (RF). For RMSE and rRMSE, the model with the largest difference between the predicted value and the true value was Lasso-LARS (RMSE = 87.45 kg/acre, rRMSE = 44.38%), while the model with the lowest was RF (RMSE = 78.46 kg/acre, rRMSE = 39.81%) (Figure 7).

(5) $$\mathrm{Yiel}d_{(s\mathrm{ummer})}=-0.3\;\times \;\mathrm{Aspect}+306.4\;\times \;\mathrm{MCARI}+1186\;\times \;\mathrm{IPVI}\;-\;792.91$$

In the study of autumn tea yield estimation, we collected 182 samples and randomly divided them into a training set (n = 127) and a validation set (n = 55). In the process of modeling, key feature variables such as OSAVI, aspect, slope, elevation, and DVI were selected, and six regression models were constructed based on these features to predict the yield of autumn tea. After the model training was completed, the performance of the model was evaluated using the validation set. In particular, we analyzed the relationship between autumn tea yield and various characteristic variables through the multiple linear regression model constructed by Equation (6). The results showed that the voting regression model performed best in the estimation of autumn tea yield, with a determination coefficient of 0.42, an RMSE of 70.6 kg/acre, and an rRMSE of 39.76% (Figure 7c).

(6) $$\mathrm{Yiel}d_{\left(\mathrm{autumn}\right)}=-0.2\times \mathrm{Aspect}+3.18\times \mathrm{Slope}+582.51\times \mathrm{OSAVI}+0.17\times \mathrm{Elevation}+83.54\times \mathrm{DVI}\;-463.61$$

In estimating the annual tea yield, we divided the 193 samples collected from 2018 to 2022 into a training set and a test set according to the ratio of 7:3 for model training and verification. The results showed that the voting regression algorithm performed best among the six regression algorithms tested, with a coefficient of determination of 0.47 and a relative root mean square error (rRMSE) of 34.62% (Figure 7d). The rRMSE range of the other five regression algorithms was between 34.62% and 38.35%. The model based on multiple linear regression is shown in Equation (7).

(7) $$\mathrm{Yiel}d_{\left(\mathrm{annual}\right)}=-1.24\times \mathrm{Aspect}+8.11\times \mathrm{Slope}+2050.16\times \mathrm{MCARI}+0.95\times \mathrm{Elevation}\;-1040.88$$

In the estimation of tea yield in different periods, the annual estimation effect was the most ideal, and the relative root mean square error was between 34.62% and 38.35%, followed by the estimation results for summer. Figure 7 shows the scatter-plot distribution of the best estimation model of fresh tea yield for the different seasons. Regardless of the year or season, particularly summer and autumn, the model generally showed underestimation in high-yield regions (above 300 kg/acre). This discrepancy may stem from the model’s inadequate capture of the intricacies associated with high-yield areas or its limited generalization ability due to a scarcity of samples from such extreme cases.

## 4. Discussion

### 4.1. Comparison of Regression Techniques for Fresh Tea Yield in Different Seasons

Before comparing the estimation models of fresh tea yield in different seasons, a significant contribution of this paper lies in the feature selection process conducted through variable importance ranking and Pearson correlation analysis. The objective of feature selection is to eliminate the multicollinearity among multiple predictors and identify the most valuable remote sensing indicators. Previous studies have demonstrated that vegetation indices [8,17,64], topographic features [65], and meteorological variables [18,66] are crucial factors for crop yield estimation. This study specifically focused on the importance of vegetation indices and topographic features in tea yield modeling. Our research results indicated that the indices including MCARI, OSAVI, EVI, GNDVI, and topographic features are important indicators for fresh tea yield estimation, as in previous research on crop yield estimation [65,67,68,69]. In future research, the tea yield estimation model will be extended to larger-scale application scenarios. To further enhance the accuracy of tea yield prediction, it is recommended to consider incorporating additional variables, such as texture features [70] and meteorological factors [71]. Furthermore, the collection of extensive field data on a large scale will contribute to enriching tea yield samples across different regions, and the applicability and portability of the fresh tea yield estimation model need to be further validated.

The accuracy of five popular machine learning algorithms, including random forest regression, voting regression, gradient boosting regression, AdaBoost regression, and Lasso-LARS regression, in predicting fresh tea yield was assessed in this study. They were compared with traditional linear regression models. Our results demonstrated the superior performance of the random forest regression model in estimating fresh tea yield during spring and summer, with R^2^ values of 0.455 and 0.5, respectively, which were significantly higher than those obtained by the other methods. In contrast, the traditional linear regression model exhibited limited capability in capturing the intricate relationship between nonlinear variables, as evidenced by its lower R^2^ values of only 0.17 and 0.38 for these two seasons. Similar findings have been obtained in other crop studies involving wheat, corn, and potato cultivation. Notably, the RF algorithm has been shown to possess distinct advantages in addressing nonlinear tasks while maintaining a relative root mean square error ranging from 5.8% to 16.7% [72]. These findings provide compelling evidence supporting the effectiveness of random forests in predicting agricultural yields, particularly when confronted with high-dimensional and diverse remote sensing data [73]. In the estimation of fresh tea yield in autumn and the whole year, the voting regression showed better performance, with an R^2^ of 0.42 for autumn and an R^2^ of 0.47 for the whole year. In contrast, the R^2^ value of random forest was 0.36 for autumn and 0.41 for the whole year. By integrating multiple models, voting regression enhances prediction accuracy and mitigates the risk of overfitting [74]. This approach also shows similar advantages in the yield estimation of other crops. For example, a study has shown that in the prediction of winter wheat yield, the R^2^ value of the voting regression algorithm reaches 0.9, which is significantly better than the performance of a single model [75]. In addition, the performance of the AdaBoost regressor and the gradient boosting regressor remains stable in different seasons, with an average relative root mean square error of 41%, which indicates that they can maintain stable accuracy when dealing with complex seasonal and environmental data. Previous studies have demonstrated the remarkable robustness of the AdaBoost regressor in estimating tea yield, achieving an RMSE value of 0.135 t/ha [9]. Compared to other methods, multiple linear regression exhibits relatively low R^2^ values across all seasons, with an average R^2^ of 0.3, which is significantly inferior to those of the remaining five regression models. Specifically, in the prediction of fresh tea yield during summer, multiple linear regression yielded an R^2^ value of 0.38, indicating a decrease in accuracy by approximately 24% compared to random forest regression. Although it has lower accuracy, multiple linear regression requires a smaller sample size and is suitable for small data sets, which is of great significance in research with limited resources [76]. Furthermore, multiple linear regression models are simple in structure and easy to implement and interpret, without the need for complex hyperparameter tuning or large amounts of computational resources [77]. Moreover, when a model is migrated to other research areas, there is no need to reconstruct the sample or retrain the model, meaning it has high versatility and computational efficiency.

### 4.2. Limitations and Strengths

Numerous researchers have been exploring different techniques to estimate tea yield. Compared with previous studies, our approach has unique characteristics. Firstly, this study obtained highly refined tea yield data for farmers’ parcels, which laid a foundation for the study of tea yield estimation based on the field scale. Secondly, we established multiple models for estimating tea yield during different seasons and throughout the entire year to facilitate comparative analysis.

However, there were several limitations in this study. Firstly, the impact of meteorological data on tea yield estimation was not considered. Previous studies have demonstrated a significant causal relationship between climate factors and tea yield [18,78,79]. When the monthly average temperature exceeds 26.6 °C, tea production will decline sharply, indicating that temperature increase has a negative impact on tea production [2]. Additionally, Merker et al. [80] found that an increase in the frequency of precipitation-free days within a given month exerts a detrimental influence on tea production. However, due to the limited size of the experimental area in this study, minimal variation was observed in meteorological conditions within the experimental area. As a result, meteorological factors were not incorporated into the yield estimation model for detailed analysis. Secondly, our study did not account for variations in prediction across different regions. Previous research has highlighted that tea yield varies significantly across seasons and locations [81]. Han et al. [82] discovered that regional differences can impact the accuracy of yield predictions. However, in our study, the tea planting area was limited to a small local area, with little change in climate, soil, and management conditions. Therefore, we did not consider the impact of regional differences on prediction performance. In future studies, it will be necessary to collect more samples from different research sites, encompassing different tea varieties and tea tree ages, to comprehensively evaluate the robustness and transferability of the tea yield estimation model. Thirdly, the source data, such as remote sensing resolution, spectral saturation phenomena, and human activities, will influence the prediction ability of machine learning. In addition, in statistical models, black-box models have limitations in explaining results, and a sufficiently large dataset is required to achieve acceptable prediction within learning models. It is hoped that the uncertainty of the model can be improved with the comprehensive collection of data and an increase in the number of input variables.

## 5. Conclusions

In conclusion, this study mainly used the spectral features of satellite images and topographic features extracted from UAV images to estimate the tea yield of Yixiang Town. We established tea yield estimation models for spring, summer, and autumn and for the whole year based on the field scale. The results showed that, at the field scale, the annual fresh tea yield estimation model performed best. Using the voting regression algorithm, the root mean square error (RMSE) was 168.7 kg/acre and the relative root mean square error (rRMSE) was 34.62%. Second best was the summer fresh tea yield model. The random forest regression algorithm performed best, with an RMSE of 78.46 kg/acre and an rRMSE of 39.81%. Based on these results, it can be concluded that the combination of spectral features extracted from satellite images and topographic features extracted from UAV images has high application potential in estimating tea yield in different seasons in Yixiang Town. However, this method results in a certain degree of overestimation or underestimation at the field scale in different seasons. In general, the tea yield estimation model based on Sentinel-2 images has potential application value, but it still needs to be further optimized to improve its prediction accuracy in different seasons and at different scales. Future research can consider using higher-resolution images and introducing more influencing factors, such as climatic conditions, soil types, and irrigation methods, to enhance the comprehensiveness and adaptability of the model.

## Figures and Tables

**Table 1:** Vegetation indices calculated from the satellite data.

VI	Name	Formula	Reference
DVI	Difference Vegetation Index	$\mathrm{DVI}=\rho_{\mathrm{NIR}}-\rho_{\mathrm{RED}}$	[29]
EVI	Enhanced Vegetation Index	$\mathrm{EVI}=2.5\times \frac{\rho_{\mathrm{NIR}}-\rho_{\mathrm{RED}}}{\rho_{\mathrm{NIR}}+6\times \rho_{\mathrm{RED}}-7.5\times \rho_{\mathrm{BLUE}}+1}$	[30]
NDVI	Normalized Difference Vegetation Index	$\mathrm{NDVI}=\frac{\left(\rho_{\mathrm{NIR}}-\rho_{\mathrm{RED}}\right)}{\left(\rho_{\mathrm{NIR}}+\rho_{\mathrm{RED}}\right)}$	[31]
RVI	Ratio Vegetation Index	$\mathrm{RVI}=\frac{\rho_{\mathrm{NIR}}}{\rho_{\mathrm{RED}}}$	[32]
SAVI	Soil-Adjusted Vegetation Index	$\mathrm{SAVI}=\frac{1.5\times (\rho_{\mathrm{NIR}}-\rho_{\mathrm{RED}})}{(\rho_{\mathrm{NIR}}+\rho_{\mathrm{RED}}+0.5)}$	[33]
MTCI	MERIS Terrestrial Chlorophyll Index	$\mathrm{MTCI}=\frac{\left(\rho_{\mathrm{RE}2}-\rho_{\mathrm{RE}1}\right)}{(\rho_{\mathrm{RE}1}-\mathrm{RED})}$	[34]
GCVI	Green Chlorophyll Vegetation Index	$\mathrm{GCVI}=\frac{\rho_{\mathrm{NIR}}}{\rho_{\mathrm{GREEN}}}-1$	[35]
REP	Red-Edge Position Index	$\mathrm{REP}=705+35\times \frac{\frac{\left(\rho_{\mathrm{RE}3}+\rho_{\mathrm{RED}}\right)}{2}-\rho_{\mathrm{RE}1}}{\rho_{\mathrm{RE}2}-\rho_{\mathrm{RE}1}}$	[36]
PVI	Perpendicular Vegetation Index	$\mathrm{PVI}=\frac{\rho_{\mathrm{NIR}}-10.489\times \rho_{\mathrm{RED}}-6.604}{\sqrt{1+10.489^{2}}}$	[29]
MCARI	Modified Chlorophyll Absorption Ratio Index	$\mathrm{MCARI}=\frac{[(\rho_{\mathrm{RE}1}-\rho_{\mathrm{RED}})-0.2\times (\rho_{\mathrm{RE}1}-\rho_{\mathrm{GREEN}})]\times \rho_{\mathrm{RE}1}}{\rho_{\mathrm{RED}}}$	[37]
OSAVI	Optimization Soil-Adjusted Vegetation Index	$\mathrm{OSAVI}=\frac{\left(\rho_{\mathrm{NIR}}-\rho_{\mathrm{RED}}\right)}{(\rho_{\mathrm{NIR}}+\rho_{\mathrm{RED}}+0.16)}$	[38]
NRI	Nitrogen Reflectance Index	$\mathrm{NRI}=\frac{\left(\rho_{\mathrm{GREEN}}-\rho_{\mathrm{RED}}\right)}{\left(\rho_{\mathrm{GREEN}}+\rho_{\mathrm{RED}}\right)}$	[39]
VARI	Visible Atmospherically Resistant Index	$\mathrm{VARI}=\frac{\left(\rho_{\mathrm{GREEN}}-\rho_{\mathrm{RED}}\right)}{\left(\rho_{\mathrm{GREEN}}+\rho_{\mathrm{RED}}-\rho_{\mathrm{BLUE}}\right)}$	[40]
$CI_{\mathrm{green}}$	Green Chlorophyll Index	$CI_{\mathrm{green}}=\frac{\rho_{\mathrm{NIR}}}{\rho_{\mathrm{GREEN}}}-1$	[41]
CVI	Chlorophyll Vegetation Index	$\mathrm{CVI}=\frac{\rho_{\mathrm{NIR}}\times \rho_{\mathrm{RED}}}{\rho_{\mathrm{GREE}N^{2}}}$	[42]
WDVI	Weighted Difference Vegetation Index	$\mathrm{WDVI}=\rho_{\mathrm{NIR}}-(1.24\times \rho_{\mathrm{RED}})$	[43]
TVI	Transform Vegetation Index	$\mathrm{TVI}=60\times (\rho_{\mathrm{NIR}}-\rho_{\mathrm{GREEN}})-100\times (\rho_{\mathrm{RED}}-\rho_{\mathrm{GREEN}})$	[44]
GNDVI	Green Normalized Difference Vegetation Index	$\mathrm{GNDVI}=\frac{\left(\rho_{\mathrm{NIR}}-\rho_{\mathrm{GREEN}}\right)}{\left(\rho_{\mathrm{NIR}}+\rho_{\mathrm{GREEN}}\right)}$	[44]
IPVI	Infrared Percentage Vegetation Index	$\mathrm{IPVI}=\frac{\rho_{\mathrm{NIR}}}{\rho_{\mathrm{NIR}}+\rho_{\mathrm{RED}}}$	[45]
MSR	Modified Simple Ratio	$\mathrm{MSR}=(\frac{\rho_{\mathrm{NIR}}}{\rho_{\mathrm{RED}}}-1)÷\sqrt{(\frac{\rho_{\mathrm{NIR}}}{\rho_{\mathrm{RED}}}+1)}$	[46]
MSAVI	Modified Soil-Adjusted Vegetation Index	$\mathrm{MSAVI}=\frac{2\rho_{\mathrm{NIR}}+1-\sqrt{(2\rho_{\mathrm{NIR}}+1{)}^{2}-8(\rho_{\mathrm{NIR}}-\rho_{\mathrm{RED}})}}{2}$	[47]
RDVI	Renormalized Difference Vegetation Index	$\mathrm{RDVI}=\frac{\rho_{\mathrm{NIR}}-\rho_{\mathrm{RED}}}{\sqrt{\mathrm{NIR}+\mathrm{RED}}}$	[48]
WDRVI	Wide Dynamic Range Vegetation Index	$\mathrm{WDRVI}=\frac{0.1\times \rho_{\mathrm{NIR}}-\rho_{\mathrm{RED}}}{0.1\times \rho_{\mathrm{NIR}}+\rho_{\mathrm{RED}}}$	[49]

**Table 2:** Parameter sets for finding optimal values for each model.

Algorithm	Hyperparameters	Parameter Sets of Candidate Values
AdaBoost	n_estimators	50, 100, 200, 300, 400
	learning_rate	0.01, 0.05, 0.1, 0.2, 0.5, 1.0
Lasso-LARS	alpha	0.01, 0.1, 1.0, 10, 100
	max_iter	100, 500, 1000, 2000
RF	n_estimators	50, 100, 200, 300, 400
	max_depth	10, 20, 30, 40, 50
	min_samples_split	2, 5, 10, 15, 20
	min_samples_leaf	1, 2, 4, 6, 8
Gradient Boosting Regressor	n_estimators	50, 100, 200, 300
	learning_rate	0.01, 0.05, 0.1, 0.2
	max_depth	3, 5, 7, 10
	min_samples_split	2, 5, 10
	min_samples_leaf	1, 2, 4

## Data Availability

The data underlying this article will be shared on reasonable request to the corresponding authors. The data are not publicly available due to Various data sources have been introduced in the paper.
